# Researching language learning motivation: A concise guide

**DOI:** 10.3389/fpsyg.2022.1107594

**Published:** 2023-01-13

**Authors:** Ran Pei, Zhiwu Zhang

**Affiliations:** School of Foreign Studies, Changsha University of Science and Technology, Changsha, China

**Keywords:** language learning, motivation, engagement, L2 Motivational Self System, emotional engagement

Language learning motivation has long been embraced by researchers as a crucial concept in second language acquisition and has arrested the attention of scholars worldwide for over six decades (Al-Hoorie, 2017; Ushioda, 2019). Among the explorations of what motivation is and how to make the best of it for language learning, scholars have centered on Gardner's socio-educational model (Gardner, 2010) and the L2 Motivational Self System (Dörnyei, 2005). The former features social–psychological views toward social group motivations and contextual variables (Dörnyei and Ryan, 2015), while the latter represents a cognitive-situated lens to learner-based classroom-oriented research. However, as the field starts to shift toward sociodynamic perspectives (Dörnyei and Ryan, 2015), what needs to be investigated is how the limitations described in Al-Hoorie's (2017) study have been addressed, and what the recent developments and future directions are. The gap has been well-filled by the book under review, *Researching Language Learning Motivation: A Concise Guide* (Al-Hoorie and Szabó, 2022), which serves as a timely, accessible, and detailed extension on the forward-pointing survey of emerging themes illustrated by Al-Hoorie (2017), and offers guidance for future research directions.

This volume contains an introduction, an afterword, and 21 chapters organized under five overarching themes. Part 1 (Chapters 1–3) addresses general reflections on the field of motivation. Ema Ushioda devotes Chapter 1 to the pedagogical implications of “motivational teaching practice.” In Chapter 2, Matthew E. Poehner adopts the notion of “perezhivanie” from sociocultural theory to reflect on the dialectical views of motivation. Similarly, Ofelia García in Chapter 3 goes beyond the psychological paradigm and advocates a sociological paradigm to unpack how language scholars have been accustomed to viewing language learners from a deficit view in terms of native speaker-like language abilities and proficiency, and proposes a translanguaging perspective to reconsider language learners in terms of their meaning-making repertoire.

Part 2 (Chapters 4–7) focuses on the critical issues of language engagement. Sarah Mercer illustrates a tripartite model of classroom engagement in great length to underline the complexities in language classrooms and ends the chapter by outlining three promising directions for future research. In Chapter 5, Phil Hiver unpacks the multi-dimensionality of engagement and illustrates teachers' adaptive expertise in managing the unpredictability in instructional settings. In Chapter 6, Alastair Henry examines the nature of language learners' goals and illustrates how quality goals can sustain motivation. Chapter 7 provides a concise overview of self-determination theory, together with its six interdependent mini-theories, and elaborates on how it contributes to motivation and engagement through dialogic interactions between teachers and students.

Part 3 (Chapters 8–11) explores motivation from “selves approaches.” Chapter 8 by Peter D. MacIntyre presents an overview of the L2 Motivational Self System and explains how it evolves from the self-theory. In Chapter 9, Mostafa Papi endeavors to test the L2 Motivational Self System by highlighting how it can be adapted to empower language teaching practice in instructional settings. In Chapter 10, Amy Thompson focuses on her experience of learning a Language other than English (LOTE) in rural America and explains how the projection of an ideal L2 self helps to maximize learning potential, regardless of limited language learning resources. In a similar vein, Chapter 11 examines the influence of technology use on L2 selves and analyzes how this technology-empowered digital visualization of L2 selves contributes to learning engagement and second language acquisition.

Part 4 (Chapters 12–15) explores studies that link motivation with emotions and affect. Through a combination of personal and historical perspectives, Jean-Marc Dewaele traces in Chapter 12 the origin and development of emotion research in the field of second language acquisition. Chapter 13, centering around emotional engagement and emotional distractions in foreign language classrooms, unveils the causes of silent L2 learners' social anxiety in light of cognitive-behavioral theory (CBT) and proposes CBT-based classroom activities to promote students' emotional engagement. Chapter 14 investigates motivation contagion through an examination of the dynamic interplay of language teachers' and learners' motivation and explains how language teachers and learners can capitalize on it and maximize its reciprocal impact. Following a detailed introduction about directed motivational currents (DMCs), emotional contagion, and group emotion, the author in Chapter 15 discusses how group-level effects can help to understand DMCs and long-term motivations.

Part 5 (Chapters 16–21) extends the exploration of motivation into newly emerging topics in the field. In Chapter 16, Ali H. Al-Hoorie and Phil Hiver highlight the need to upgrade and diversify the current toolbox of methods for conducting complex dynamic systems theory (CDST) research. Based on a longitudinal study of nine Indonesian learners' motivation in learning English, Chapter 17 delves into “elite multilingualism” through notions of desires, identities, and power from a critical discourse analysis perspective. Chapter 18 expounds on the linguistic and cultural diversities in a globalized world of ever-frequent migration and mobility and ventures to investigate motivation in multi-ethnic settings. In Chapter 19, Zana Ibrahim elaborates on the liberating effect of the concept of English as a Lingua Franca (ELF) on second language learners. Based on his rich experience in applied linguistics and neuroscience, Robert S. Murphy dedicates Chapter 20 to seven neuroELT maxims that can lead to success in motivating students in language classrooms. Finally, Chapter 21, by integrating anthropological, affective, and social perspectives, sheds light on the effects of group dynamics on learners' wellbeing and language learning motivation.

Overall, this volume, with its chapters contributed by established scholars and active researchers worldwide, features succinct and accessible overviews for newcomers to the field of language learning motivation. For both students and novice researchers, the current volume could be a good start to embark on the journey of language learning motivation, as its comprehensive and up-to-date overviews can offer the readership an encompassing view of the major approaches and topics in researching language learning motivation. In view of the approaches, while the foundational social–psychological approach featuring integrative motivation and instrumental motivation can be found mentioned in various chapters, its applicability is being challenged in various language learning scenarios, where English as a Lingua Franca (Chapter 19) or multilingualism (Chapter 18) is widely accepted. Under both circumstances, the monolingual bias has to be abandoned and the integrative motivation may no longer apply, as learners begin to abandon the deficit view of achieving native-like proficiency and embrace the learning resources that come with their first language; they view their linguistic and cultural diversity as a blessing to foster their multilingual ideal self-aspirations rather than a burden for an ideal English self. Another challenge the social-psychological approach confronts is its deterministic view of considering motivation as an individual-difference factor, which does not help to explain the dynamic change of motivation. This is addressed methodologically in Chapter 16 by drawing on conceptualizations from the complex dynamic systems theory. Second, the cognitive-situated approach, which incorporates cognitive theories in language learning motivation in specific learning contexts, can be found in classroom-based studies in this volume. For instance, the self-determination theory (Chapter 7) is outlined as a general theory of motivation and illustrates the dialogic nature of how language learners more involved in decision-making processes can influence their motivation and engagement within their learning environment. Third, the sociodynamic approach, which considers motivation development in a non-linear fashion and within an interplay of multiple personal, social, and contextual factors, is exemplified in Part 3. Taking Chapter 9 for example, the L2 Motivational Self System is elaborated and relevant studies are reviewed to explicate how L2 selves can be explored in motivating language learners in the classroom context.

As for motivation Research Topics, the contributions in this volume have presented a good summary of well-researched motivation topics as well as insightful guidance for future research directions. For example, not only are motivation and engagement explored from multiple perspectives, such as the sociocultural theory (Chapter 2), the goal self-concordance theory (Chapter 6), and the cognitive-behavioral theory (Chapter 13), but also emotions and affect are also incorporated in the probing of language learning motivation in Part 4. As positive psychology takes a firm hold within second language acquisition, researchers have shifted their attention from negative emotional dimensions to positive emotions (Dewaele et al., 2019). As motivation researchers begin to equip themselves with new lenses of positive emotions, they can reorientate what they can achieve from creating positive emotions in motivating language learners. Readers can also find inspiration from Part 5, where newly emerging topics are introduced to guide future research. For instance, language learning within the contexts of English as a Lingua Franca (Chapter 19) or plurilingualism (Chapter 18) creates new opportunities in researching language learners' motivation. These new changes bring with them new language learning goals and appraisals of their language proficiency, leading to language learners' different views of looking at language learning obstacles and challenges as well as their own competencies in language learning. Therefore, they can exert a profound impact on language learners' overall motivation, and this less-traveled territory can present a new direction for those who are interested.

While the contributions to this volume can be applauded, there is still room for improvement. For instance, emotional engagement, which is explored in Chapter 13 in Part Four, could be incorporated in Part Two, which addresses topics related to language engagement; Chapter 21 in Part Five could also be included in Part Four, as class group dynamics bears resemblance to group interactions and emotions in the L2 classroom. This being said, it is understandable that the editors, when faced with book chapters that may straddle two sections, make their decisions by weighing the central focus of those chapters.

With these reservations aside, this concise guide, with its international expertise, well-balanced presentation of overviews and emerging topics, as well as its accessible and approachable style, can be a springboard for students and researchers interested in language learning motivation. For postgraduates, it can offer a perspicacious overview of the historical development of the field and provide them with established theories and views; for researchers, the emerging topics and the untrodden territories listed in the afterword by Lourdes Ortega could be enlightening to expand their research within language learning motivation.

## Author contributions

RP wrote the initial draft of the article. ZZ provided several rounds of critical and constructive feedback to the drafts, and added comments and analysis. The final draft is the result of RP and ZZ's collective efforts. All authors contributed to the article and approved the submitted version.
